# Effects of RNAi-Mediated Knockdown of Histone Methyltransferases on the Sex-Specific mRNA Expression of *Imp* in the Silkworm *Bombyx mori*

**DOI:** 10.3390/ijms15046772

**Published:** 2014-04-22

**Authors:** Masataka G. Suzuki, Haruka Ito, Fugaku Aoki

**Affiliations:** Division of Biological Sciences, Department of Integrated Biosciences, Graduate School of Frontier Sciences, the University of Tokyo, 302 Bioscience-Bldg, 5-1-5 Kashiwanoha, Kashiwa, Chiba 277-8562, Japan; E-Mails: itofami@f6.dion.ne.jp (H.I.); aokif@k.u-tokyo.ac.jp (F.A.)

**Keywords:** alternative splicing, DOT1L, histone H3 methylation, sex determination

## Abstract

Sexual differentiation in *Bombyx mori* is controlled by sex-specific splicing of *Bmdsx*, which results in the omission of exons 3 and 4 in a male-specific manner. In *B. mori*, insulin-like growth factor II mRNA-binding protein (Imp) is a male-specific factor involved in male-specific splicing of *Bmdsx*. Male-specific *Imp* mRNA results from the male-specific inclusion of exon 8. To verify the link between histone methylation and alternative RNA processing in *Imp*, we examined the effects of RNAi-mediated knockdown of several histone methyltransferases on the sex-specific mRNA expression of *Imp*. As a result, male-specific expression of *Imp* mRNA was completely abolished when expression of the H3K79 methyltransferase DOT1L was repressed to <10% of that in control males. Chromatin immunoprecipitation-quantitative PCR analysis revealed a higher distribution of H3K79me2 in normal males than in normal females across *Imp*. RNA polymerase II (RNAP II) processivity assays indicated that RNAi knockdown of *DOT1L* in males caused a twofold decrease in RNAP II processivity compared to that in control males, with almost equivalent levels to those observed in normal females. Inhibition of RNAP II-mediated elongation in male cells repressed the male-specific splicing of *Imp*. Our data suggest the possibility that H3K79me2 accumulation along *Imp* is associated with the male-specific alternative processing of *Imp* mRNA that results from increased RNAP II processivity.

## 1. Introduction

Alternative splicing of pre-mRNA is an essential mechanism in the regulation of differential gene expression that can produce functionally distinct proteins from a single gene based on the developmental or physiological state of the cells in a multicellular organism [1,2]. Recent studies estimate that 90% of human genes are alternatively spliced [3], and several thousand different mRNA isoforms can be produced from a single gene. Although many examples describe how alternative splicing regulates gene expression, the mechanisms involved are less well understood [4–8].

Alternative splicing is thought to be regulated by the interaction of splicing factors and splicing enhancers (or silencers). Alternative splicing regulatory mechanisms have been investigated, structural models of spliceosomes have been proposed, and many RNA regulatory elements have been characterized; however, the emerging complexity of alternative splicing regulation suggests that these approaches have not sufficiently described how alternative splicing is regulated. Recent provocative studies point to a key function of chromatin structure and histone modification in alternative splicing regulation [9–11]. For example, the nucleosome occupancy level was lower in cassette exons than in constitutively spliced exons [12–14]. H3K36me3 and H3K9ac were related to the exon-skipping event of NCAM [15] and the levels of H3K36me3 differed in mutually exclusive exons of several genes among different cell types. Moreover, a genome-wide study across different species revealed that H3K36me3 was depleted in skipped exons [13,16]. Other histone modifications such as H3K4me1, H3K4me3, H3K27me3, and H3K9me1 have been associated with the alternative splicing events of FGFR2 [17]. H3K4me3 was suggested to affect the alternative splicing events of CHD1 [18], while H3K9me3 was associated with multiple exon skipping of CD44 [19]. A recent genome-wide chromatin immunoprecipitation (ChIP)-seq analysis of histone H3 methylation in mammals revealed that alternative exons are preferentially marked with H3K4me1, H3K27me3, and H3K79me2, while being marked with H3K4me2, H3K4me3, and H3K36me3 at significantly lower levels [20].

In the silkworm *Bombyx mori*, the chromosomal sex determination mechanism is distinct from that of *Drosophila melanogaster*, with female (ZW) being the heterogametic sex and male (ZZ) the homogametic sex. The female sex in *B. mori* is determined by the presence of a dominant feminizing factor, *Feminizer* (*Fem*), on the W chromosome [21]. Note that no sex-specific regulatory *Sxl* homolog has been isolated from *B. mori* [22], and no *tra* homolog has been found in the *Bombyx* genome [23]. Despite these differences, a *B. mori dsx* homolog (*Bmdsx*) has been implicated in sex determination [24]. The primary transcript of the *Bmdsx* gene is alternatively spliced in males and females to yield sex-specific mRNAs that encode male-specific (BmDSXM) and female-specific (BmDSXF) polypeptides [25]. We found that unlike *Drosophila dsx*, the *Bmdsx* female exon is devoid of putative TRA/TRA-2 binding sites [25]. Instead, the splicing inhibitor BmPSI and a *B. mori* insulin-like growth factor II mRNA-binding protein (Imp) regulate male-specific splicing of *Bmdsx* [26,27]. *Imp* is localized on the Z chromosome and is expressed in a male-specific manner in various tissues. In male cells, the male-specific *Imp* mRNA is formed as a result of the inclusion of exon 8 and the promoter-distal poly(A) site choice, whereas non-sex-specific polyadenylation occurs at the promoter-proximal poly(A) site downstream of exon 7 [28]. The molecular mechanisms underlying the sex-specific splicing regulation of this gene remain unclear.

To verify the link between histone methylation and alternative RNA processing in *Imp* mRNA production, we investigated the effects of RNAi-mediated knockdown of several histone methyltransferases (HMTases) on sex-specific mRNA expression of *Imp*. Notably, the male-specific expression of *Imp* mRNA was completely abolished when expression of the H3K79 methyltransferase DOT1L was repressed to <10% of that in control males. Here, we provide several lines of evidence suggesting that H3K79me2 accumulation along *Imp* is associated with male-specific alternative RNA processing in *Imp* mRNA production, resulting from increased RNAP II processivity. To our knowledge, this is the first report to associate histone modification with the regulation of sex-specific alternative splicing.

## 2. Results and Discussion

### 2.1. Results

#### 2.1.1. Knockdown of *DOT1L* Abolished Male-Specific Expression of the *Imp* mRNA

**Figure 1.**
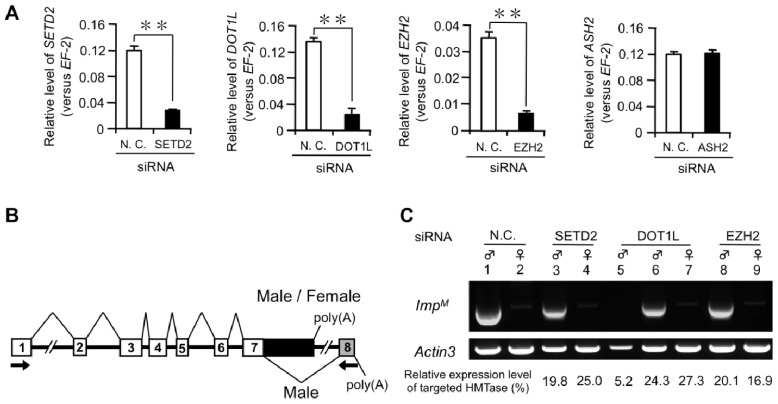
The effect of histone methyltransferase (HMTase) knockdown on sex-specific splicing of *Imp*. (**A**) Quantification of HMTase mRNA expression 4 days after siRNA injections using quantitative reverse transcription (qRT)-PCR. *Elongation factor 2* (*EF-2*) served as an internal standard. Error bar: SD; *n* = 8–24 individuals. ** *p* < 0.01, Student’s *t*-test; (**B**) A schematic diagram of alternative splicing in *Imp* pre-mRNA. Exons are numbered and displayed as boxes. The gray box indicates the male-specific exon. The V-shaped lines above and below the diagram denote the splice variants observed in males and females. *Imp* contains two poly(A) sites. The proximal promoter site located within intron 7 is utilized in a non-sex-specific manner. The distal promoter site is selected in a male-specific manner and exists near the end of exon 8. The arrows indicate the approximate location of primers used for RT-PCR in **C**; (**C**) The male-specific *Imp* mRNA (*Imp^M^*) was detected by RT-PCR and analyzed in a 1% agarose gel. The upper panel depicts expression of *Imp^M^*, and the lower panel shows the amplification of the *Actin3* transcript, which served as a positive control for RNA extraction and RT-PCR. Sex identification of each egg was performed by PCR amplification of the W-specific random amplified polymorphic DNA (RAPD) marker *Rikishi*. The expression levels of targeted HMTases relative to the negative control embryos in each individual examined are indicated below each lane.

Recent genome-wide ChIP-seq analyses revealed that alternatively spliced exons are preferentially marked with H3K4me1, H3K27me3, and H3K79me2 [20]. Furthermore, a genome-wide study across different species revealed that H3K36me3 was depleted in skipped exons [13,16]. To investigate whether these epigenetic marks are associated with male-specific splicing of *Imp* pre-mRNA, we performed RNAi knockdown of several histone methyl transferases (HMTases) such as ASH2, EZH2, SETD2, and DOT1L known to modify H3K4, H3K27, H3K36, and H3K79, respectively, in embryos. Microinjection of dsRNA into *B. mori* embryos has been used successfully in many studies, although silencing levels vary [29]. siRNAs were injected into eggs during the early embryonic stage 6–8 h after oviposition, a developmental period known to be sensitive to RNAi-mediated gene knockdown [30]. Total RNA was extracted from each egg 4 days after injection. As shown in Figure 1A, qRT-PCR confirmed a significant reduction in *EZH2*, *SETD2*, and *DOT1L* transcript levels in embryos injected with siRNAs targeting these HMTase-coding genes. Injection of *ASH2* siRNA failed to reduce the level of the target gene mRNA even though we used several siRNA sequences. Therefore, we focused on the knockdown effects of *SETD2*, *DOT1L*, and *EZH2* on the expression of the male-specific *Imp* mRNA (*Imp^M^*). RNAi knockdown of *SETD2* and *EZH2* had no influence on the expression of *Imp^M^* (Figure 1C, lanes 3, 4, 8, and 9). Notably, the expression of *Imp^M^* was completely abolished when the *DOT1L* expression level was repressed to <10% of that in control males (Figure 1C, lane 5). Five of six examined individuals whose *DOT1L* level was less than 10% also showed the disappearance of male-specific *Imp* expression. Further study is required to determine whether a similar effect on the expression of *Imp^M^* occurs when the expression levels of *SETD2* and *EZH2* are repressed <10% of that in control males.

#### 2.1.2. *DOT1L* Knockdown Affects Male-Specific Splicing of *Imp* Pre-mRNA

**Figure 2.**
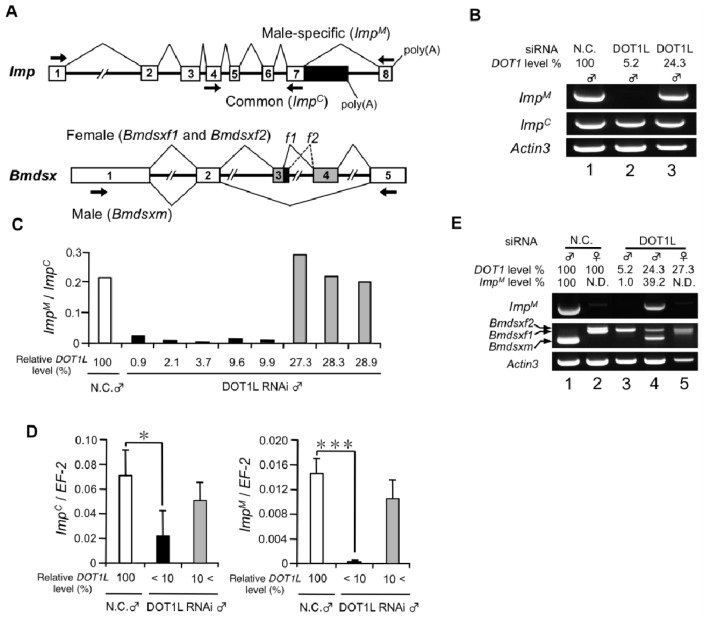
Effect of *DOT1L* knockdown on male-specific splicing of *Imp*. (**A**) The upper panel shows the location of the reverse transcription (RT)-PCR primers used for detection of *Imp* mRNA. The arrows above the diagram indicate the primers that were used for amplification of *Imp^M^*, and the arrows below the diagram show the primers used to amplify *Imp* mRNA transcribed from a region common to both sexes (*Imp^C^*). The lower panel indicates a schematic diagram of alternative splicing in the *Bmdsx* pre-mRNA. The gray boxes indicate the female-specific exons. The arrows point to the approximate locations of the primers used for RT-PCR in E; (**B**) Expression of *Imp^M^* and *Imp^C^* was detected by RT-PCR with primers illustrated in A and analyzed in a 1% agarose gel. The cDNA samples examined in lanes 1, 2, and 3 were identical to those used in lanes 1, 5, and 6, respectively, in Figure 1C. The bottom panel shows amplification of the *Actin3* transcript, which served as a positive control for RNA extraction and RT-PCR. The expression of *DOT1L* relative to the negative control embryos in each individual is indicated above each lane; (**C**) The ratio of *Imp^M^* to *Imp^C^* was analyzed by qRT-PCR; (**D**) Quantification of *Imp^C^* (left panel) or *Imp^M^* mRNA expression (right panel) by qRT-PCR. The *elongation factor 2* (*EF-2*) served as an internal standard. SD; *n* = 5 individuals. * *p* < 0.05, *** *p* < 0.001, Student’s *t*-test; (**E**) Female- or male-specific splicing of *Bmdsx* pre-mRNA was detected by RT-PCR and analyzed on a 1% agarose gel. The upper panel shows expression of *Imp^M^*, and the middle panel indicates the female- and male-specific splicing products of *Bmdsx* (*Bmdsxf1*, *Bmdsxf2* and *Bmdsxm*, respectively). The lower panel shows amplification of the *Actin3* transcript, which served as a positive control for RNA extraction and RT-PCR. The expression levels of *DOT1L* and *Imp^M^* relative to those of the negative control embryos in each individual are indicated above each lane.

The above results indicate that *DOT1L* knockdown led to the loss of male-specific *Imp* expression in males. Two possible explanations may account for this phenomenon: *DOT1L* knockdown repressed *Imp* transcription or downregulation of *DOT1L* inhibited the splicing between exons 7 and 8 in *Imp* pre-mRNA. To examine these possibilities, we performed comparative analyses of *Imp^M^* and the *Imp* transcript common to both sexes (*Imp^C^*). RT-PCR analyses using primers described in Figure 2A demonstrated that *DOT1L* knockdown had little or no effect on the *Imp^C^* mRNA expression (Figure 2B). In contrast, *Imp^M^* transcript was not detected in a male with the *DOT1L* expressed to <10% of that in a control male (Figure 2B, lane 2). Next, using qRT-PCR, *Imp^M^* mRNA expression was compared with that of the *Imp^C^* mRNA. As shown in Figure 2C, extremely low expression of *Imp^M^* mRNAs were observed in all males with *DOT1L* levels <10% of those in control males relative to *Imp^C^* mRNA. These results indicate that *DOT1L* knockdown exclusively decreased the expression of the *Imp^M^* transcript. To rule out the possibility that down-regulation of *DOT1L* by RNAi affects *Imp* RNA transcription, qRT-PCR was performed to measure the *Imp^C^* mRNA level that represents total *Imp* mRNA expression. As shown in Figure 2D, the *Imp^C^* mRNA level in *DOT1L* knockdown males with *DOT1L* expression <10% of that in control males was decreased to a level less than one-third of that in control males (Figure 2D, left panel). In contrast, a more prominent reduction in *Imp^M^* mRNA was observed in the same *DOT1L* knockdown males (Figure 2D, right panel). These results indicate that *DOT1L* knockdown affects *Imp* RNA transcription but the male-specific splicing of *Imp* is repressed more severely by DOT1 depletion, leading to the loss of the *Imp^M^* expression. These results highlight the link between DOT1L and male-specific alternative splicing of *Imp* pre-mRNA. To investigate whether the decreased level of the *Imp^M^* mRNA caused by DOT1L depletion affects sex-specific splicing of *Bmdsx*, RT-PCR analysis was performed using primers designed to allow discrimination between female- and male-specific *Bmdsx* transcripts (Figure 2A, lower panel). As shown in Figure 2E, decreased expression of *Imp^M^* induced the expression of the female-specific *Bmdsx* (*Bmdsxf1* and *Bmdsxf2*) mRNAs in addition to the male-specific *Bmdsx* (*Bmdsx^M^*) mRNA (Figure 2E, lane 4). Only *Bmdsxf1* expression was observed males whose *Imp^M^* expression was severely diminished by DOT1L depletion (Figure 2E, lane 3). These results were consistent with our previous data showing that downregulation of *Imp^M^* by RNAi in male cells increases female-specific splicing of *Bmdsx* [27]. Sometimes a doublet band was seen in the *DOT1L-*knockdown females (Figure 2E, lane 5). The upper band corresponded to *Bmdsxf1* and the lower band was expected to be a splice variant that lacks the third exon. At present we do not know exactly the reason for the appearance of the splice variant that lacks exon 3 in the *DOT1L*-knockdown female.

#### 2.1.3. High Levels of H3K79me2 Favor Inclusion of Male-Specific Exon of *Imp*

**Figure 3.**
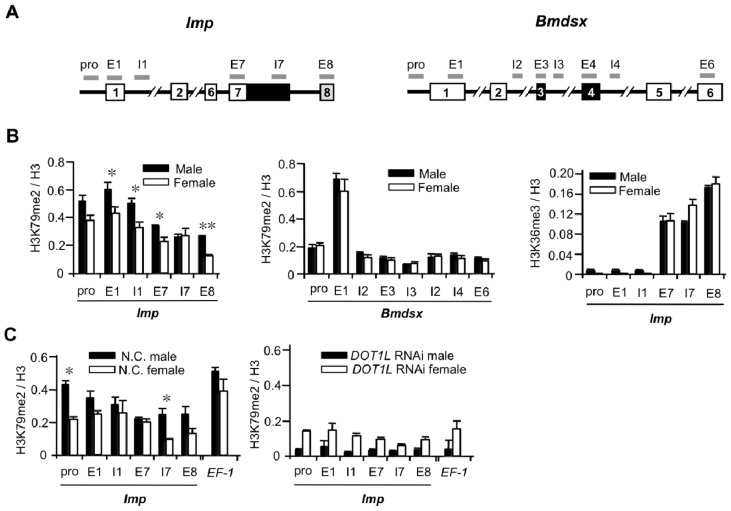
Increased H3K79 methylation across *Imp* in males. (**A**) Schematic representation of *Imp* and *Bmdsx* genes showing the distribution of quantitative (q) PCR amplicons used in the analysis; (**B**) Mapping of H3K79me2 across *Imp* (left panel) and *Bmdsx* (middle panel) and of H3K36me3 across *Imp* (right panel) in female (black) and male (white) larval tissues by chromatin immunoprecipitation (ChIP)-qPCR. Values represent the means ± SE of six qPCR values from one representative of five independent experiments. * *p* < 0.05, ** *p* < 0.01, Student’s *t*-test; (**C**) ChIP assays with antibodies to H3K79me2 and H3 and chromatin prepared from 60-pooled negative control embryos of each sex (left panel) or 60-pooled *DOT1L* siRNA-injected embryos of each sex (right panel). The relative enrichment of H3K79me2 on *EF-2* exon2 or along *Imp* was quantified by qPCR using primer sets indicated in A and expressed as a fraction of histone H3. Values represent the means ± SE of two independent qPCR assays from one representative of two independent experiments. * *p* < 0.05, Student’s *t*-test. The percentage of input was normalized to unmodified H3.

**Figure 4.**
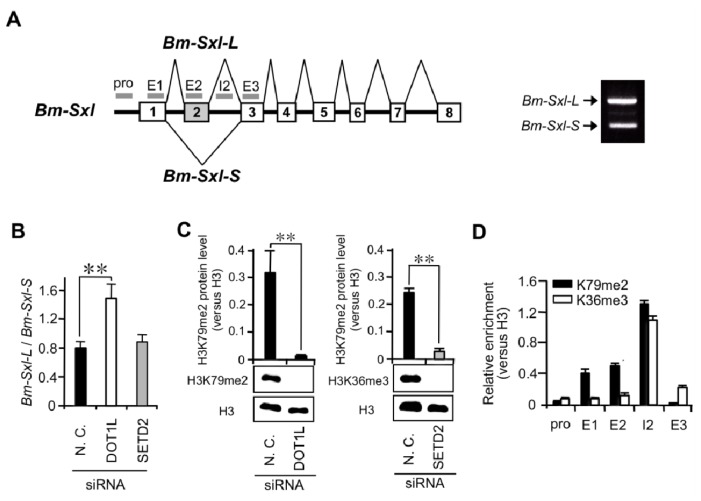
Effect of *DOT1L* knockdown on alternative splicing of *Bm-Sxl* and distribution of H3K79me2 around the alternatively spliced exon in *Bm-Sxl*. (**A**) Schematic representation of *Bm-Sxl* showing the distribution of qPCR amplicons used in the analysis; (**B**) The ratio of *Bm-Sxl-L* to *Bm-Sxl-S* was analyzed by qRT-PCR. SD; *n* = 5 individuals. ** *p* < 0.01, Student’s *t*-test; (**C**) Western blotting analysis of H3K79me2 protein extracted from negative control or *DOT1L* siRNA-injected embryos using anti-H3K79me2 and anti-H3 antibodies (lower left panel). Quantification of H3K79me2 protein levels, as detected by Western blotting analysis (upper left panel). The intensity of each band was measured using Bioimage Analyser LAS1000. H3K79me2 protein level was normalized to the H3 protein level. Values represent the means ± SE of six bands from one representative of two independent experiments. ** *p* < 0.01, Student’s *t*-test. The same analysis was performed on H3K36me3 protein extracted from negative control or *SETD2* siRNA-injected embryos using anti-H3K79me2 and anti-H3 antibodies (upper and lower right panels); (**D**) Mapping of H3K79me2 (black) and H3K36me3 (white) around *Bm-Sxl* exon 2 in larval tissues by ChIP-qPCR. Values represent the means ± SE of six qPCR values from one representative of five independent experiments.

The above results support the possibility that H3K79 methylation marks are directly or indirectly associated with the regulation of the male-specific splicing of *Imp* pre-mRNA. We next performed comparative mapping of H3K79me2 across the alternatively spliced regions of *Imp* in females and males by ChIP-qPCR. As shown in Figure 3B, higher distributions of H3K79me2 were observed in males than in females across *Imp* (Figure 3B, left panel). Although the H3K79me2 enrichments were not limited to the alternatively spliced exon 8, most of the significant differences between males and females were observed in exon 8. In contrast, no significant differences in the levels of H3K79me2 over *Bmdsx* were observed (Figure 3B, middle panel). To investigate whether the distribution of the other histone marks across *Imp* differed between males and females, we performed comparative mapping of H3K36me3 across *Imp* in females and males by ChIP-qPCR. As a result, no significant difference between males and females was observed in the levels of H3K36me3 over *Imp* (Figure 3B, right panel). To examine whether siRNA-mediated knockdown of *DOT1L* reduces the level of H3K79me2 accumulation, *DOT1L* siRNA-injected embryos were subjected to ChIP-qPCR analyses. Although higher distributions of H3K79me2 were observed in males than in females across *Imp* in negative control individuals (Figure 3C, left panel), *DOT1L* knockdown exerted a marked influence on the accumulation of H3K79me2, with an at least 10-fold reduction throughout the regions examined in males (Figure 3C, right panel). Western blotting analysis demonstrated that *DOT1L* siRNA-injection efficiently reduced the total H3K79me2 level (Figure 4C). Since Dot1 in yeast and its homologs appear to be solely responsible for H3K79 methylation [31], reduction in the accumulation of H3K79me2 across *Imp* by DOT1L depletion could be attributed to a reduction in the amount of DOT1L interacting with *Imp*. These results provide overwhelming support for the specificity of the ChIP data. However, reduced accumulation of H3K79me2 in females was less than that in males. This difference in the extent of reduction in H3K79me2 between males and females could be attributed to differences in *DOT1L*-knockdown efficiencies between males and females, as shown in Figure 1C and Figure 2E. Mean *DOT1L* expression relative to control individuals was 27.6% in *DOT1L* knockdown females and 15.1% in *DOT1L*-knockdown males. At present we cannot explain the sexual difference in knockdown efficiencies. Although loss of H3K79me2 may affect indirectly the distribution of H3K4me1, H3K27me3, or H3K36me3, such indirect effect seems unlikely to affect the male-specific splicing of *Imp* since RNAi knockdown of ASH2, EZH2, and SETD2 had no influence on the expression pattern of *Imp* mRNA (Figure 1C). Overall we conclude that methylation of histone H3 across *Imp* involves the HMTase DOT1L and high levels of H3K79me2 favor inclusion of exon 8.

To verify the link between the high distributions of H3K79me2 and inclusion of alternative exons in genes other than *Imp*, we examined the effects of *DOT1L*-knockdown on a *Bombyx* homolog of *Sex-lethal* gene (*Bm-Sxl*). The primary transcript of the *Bm*-*Sxl* gene is alternatively spliced to yield two isoforms, *Bm*-*Sxl-L* and *Bm*-*Sxl-S* [22]. *Bm*-*Sxl-L* consists of eight exons, while *Bm*-*Sxl-S* is a splice variant that lacks the second exon (Figure 4A). qRT-PCR analysis demonstrated that *Bm*-*Sxl-L*/*Bm*-*Sxl-S* ratio was more than twofold higher in *DOT1L*-knockdown embryos than in negative control embryos (Figure 4B). This result indicated that the *DOT1L*-knockdown relatively increased the inclusion of alternatively spliced exon (exon 2). In contrast, *SETD2*-knockdown caused no influence on the *Bm*-*Sxl-L*/*Bm*-*Sxl-S* ratio. Western blotting analysis demonstrated that *DOT1L* siRNA-injection and *SETD2* siRNA-injection efficiently reduced the total H3K79me2 level and total H3K36me3 level, respectively (Figure 4C). To investigate whether the high distributions of H3K79me2 is correlated with DOT1L-dependent inclusion of *Bm-Sxl* exon 2, we performed mapping of H3K79me2 around the exon 2 by ChIP-qPCR. The H3K79me2 mark was specifically enriched on the regions around exon 2 as compared with the H3K36me3 mark (Figure 4D). These results suggest that enrichment of H3K79me2 could be correlated with DOT1L-dependent inclusion of alternative exons.

#### 2.1.4. Male and Female Differences in RNAP II Processivity in the *Imp* Gene

**Figure 5.**
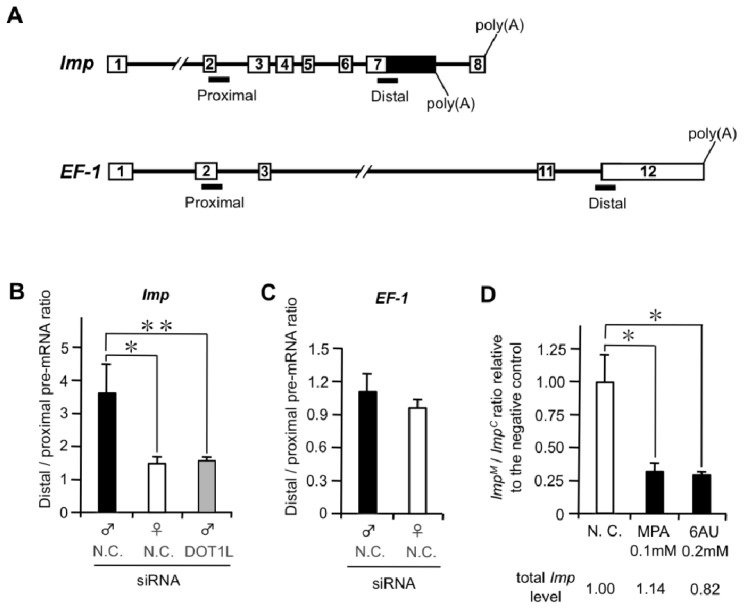
Male and female differences in RNAP II processivity in *Imp*. (**A**) Schematic diagram of *Imp* and *EF-1* showing the distribution of the proximal and distal amplicons (black bars) used for qPCR analysis; (**B**,**C**) RNAP II processivity was determined as a ratio of the proximal and distal pre-mRNA sequences (distal/proximal) of *Imp* (**B**) and *EF-1* (**C**). The abundance of each pre-mRNA was determined by quantitative (q)PCR. Values represent the means ± SE of six qPCR values from three individuals; (**D**) Inhibitors of nucleotide biosynthesis suppress male-specific splicing of *Imp* pre-mRNA. qRT-PCR analysis was performed to calculate the ratio of *Imp^M^* to *Imp^C^*. The *Imp^M^*/*Imp^C^* ratio in male cells treated with 0.1 mM mycophenolic acid (MPA) or with 0.2 mM 6-azauracil (6AU) (**C**) is relative to that in the negative control cells in each experiment. Values represent the means ± SE from three individual experiments. * *p* < 0.05, ** *p* < 0.01, Student’s *t*-test. Values below each graph indicate total *Imp* mRNA expression in the cells examined relative to those in the negative control cells.

Our data suggest a link between male-specific alternative splicing of *Imp* pre-mRNA and higher accumulation of H3K79me2. H3K79me2 modification is tightly associated with active transcription [32–34]. Moreover, several observations have suggested a close relationship between RNAP II dynamics and alternative splicing [35,36]. Based on these observations, we analyzed whether RNAP II processivity in *Imp* differs between males and females. To this end, we utilized an observation previously identified by others that transcription by a slower mutant RNAP II results in an increase in the ratio between promoter-proximal and promoter-distal pre-mRNA [15,36]. qRT-PCR of *Imp* pre-mRNA with primer sets located at each end of *Imp* (Figure 5A) showed that the distal/proximal (D/P) pre-mRNA ratio was more than twofold higher in males than in females (Figure 5B). In contrast, no significant difference in the D/P ratio was observed in the control gene, *B. mori elongation factor-1* (*EF-1*) (Figure 5C). These data suggest that a link between male-specific alternative splicing of *Imp* pre-mRNA and higher RNAP II processivity at this locus. As shown in Figure 5B, *DOT1L* knockdown reduced the D/P ratio in the male to a level similar to that in the normal female. Taken together with the ChIP-qPCR data indicated in Figure 3C, this result supports the possibility that higher distribution of H3K79me2 across *Imp* results in increased RNAP II processivity at this locus.

#### 2.1.5. Suppression of Male-Specific *Imp* Pre-mRNA Splicing by Inhibitors of Nucleotide Biosynthesis

Sensitivity to MPA and the base analog 6AU are hallmarks of transcription elongation defects in yeast [37–39]. Both drugs cause the depletion of cellular nucleotide substrate pools required by RNA polymerases [40,41]. *In vitro*, RNAP II complexes pause and arrest more frequently under conditions of limiting amounts of nucleotides [42], suggesting that the *in vivo* hypersensitivity to these compounds is due to increased dependence on factors that promote elongation by RNAP II. Treatment with these drugs causes inhibition of exon skipping [36,43] because the extent of skipping of alternative exons correlates with the elongation rate of RNAP II [36]. Based on these reports, we investigated the effects of these inhibitors on male-specific splicing of *Imp* pre-mRNA. Male cultured cells (NIAS-Bm-M1) that were not growth-arrested were treated with these inhibitors at concentrations established previously [43]. The efficiency of male-specific splicing was estimated by the ratio of *Imp^M^*/*Imp^C^* mRNA. The *Imp^M^*/*Imp^C^* ratio in male cells treated with each drug relative to that in the negative control cells is indicated in Figure 5D. In the MPA experiment, an approximately threefold decrease in the *Imp^M^*/*Imp^C^* ratio was observed. A similarly high reduction in the *Imp^M^*/*Imp^C^* ratio was observed when the male cells were treated with 6AU. Importantly, no significant difference was detected in the level of total *Imp* mRNA between control cells and inhibitor-treated cells, indicating that the reduction in the *Imp^M^*/*Imp^C^* ratio represented simply the reduction in male-specific *Imp* mRNA expression. Taken together with measurements of RNAP II processivity in Figure 5B, the simplest explanation for these results is that drug-induced reduction in transcript elongation led to the inhibition of the male-specific splicing of *Imp* pre-mRNA.

#### 2.1.6. Embryonic Lethality Caused by *DOT1L* Knockdown

Above results indicated that *DOT1L* knockdown caused decreased expression of *Imp^M^*, leading to the expression of the female-specific *Bmdsx* mRNA in male embryos (Figure 2E). To evaluate whether *DOT1L* does indeed play an important role in sexual differentiation, we investigated the effect of *DOT1L* knockdown on the development of sexual phenotypes. The highest hatch rate of the control siRNA-injected embryos in six trials was 21.7% (Table 1), which was still lower than that reported by another group [30]. Presumably, this difference was caused by technical issues related to microinjection. Similarly low hatchability in negative control dsRNA-injected eggs is reported by the other group [44]. Compared with the control embryos, nearly all the embryos injected with *DOT1L* siRNA did not hatch, suggesting embryonic lethality (Table 1). One hatched larva was obtained from a male egg injected with *DOT1L* siRNA that survived to the adult stage. This male had normal fertility and its genital organs showed no abnormalities when viewed under a dissecting microscope (data not shown).

### 2.2. Discussion

Imp in *B. mori* has been identified as a male-specific RNA-binding protein involved in the regulation of male-specific splicing of *Bmdsx* [27]. The pre-mRNA of *Imp* undergoes sex-specific RNA processing. In male cells, male-specific *Imp* mRNA contains exon 8 and the distal promoter poly(A) site choice, whereas non-sex-specific polyadenylation occurs at the proximal promoter poly(A) site downstream of exon 7. Here, we found that *DOT1L* knockdown affects male-specific splicing of *Imp* pre-mRNA, leading to loss of male-specific *Imp* expression (Figure 2B,C). In support of this result, higher distributions of H3K79me2 were observed in males than in females or in *DOT1L* knockdown males across *Imp* (Figure 3B,C). Strong link between enrichment of H3K79me2 and DOT1L-dependent inclusion of alternative exons was also observed in *Bm-Sxl* (Figure 4). Comparative analysis of RNAP II processivity indicated that higher distribution of H3K79me2 across *Imp* was correlated to increased RNAP II processivity at this locus (Figure 5B). Inhibition experiments using inhibitors of RNAP II elongation suggested that the higher elongation rate was tightly associated with male-specific RNA processing of *Imp* pre-mRNA (Figure 5D). Together, our data suggest that H3K79me2 accumulation along *Imp* is associated with male-specific alternative RNA processing in *Imp* mRNA production, resulting from increased RNAP II processivity.

The alternative RNA processing pattern of *Imp* pre-mRNA closely resembles that found in *Drosophila polo* pre-mRNA. *Polo*, which is a cell cycle gene, also contains a proximal and a distal poly(A) site in the 3′untranslated region (UTR) to produce alternative mRNA that differ in their 3′UTR length [45]. In a mutant *Drosophila* strain that displays a reduced RNAP II elongation rate, RNAP II occupancy along *polo* is altered and the proximal poly(A) site is used 3.5-fold more efficiently than in wild-type flies [46]. An increase in proximal poly(A) site usage was also observed in five other alternatively polyadenylated transcripts in *Drosophila*. These results in *Drosophila* show that the kinetics of RNAP II can determine alternative poly(A) site selection. As shown in Figure 5D, significant reduction in the *Imp^M^*/*Imp^C^* ratio was observed when the male cells were treated with inhibitors of RNAP II elongation. This result indicates that drug-induced repression of transcript elongation leads to the reduction in utilization of distal poly(A) site, resulting in the relative increase in proximal poly(A) site usage. Slow RNAP II presumably exposes the proximal poly(A) site on the nascent transcript to the polyadenylation machinery for a longer time before RNAP II transcribes the distal poly(A) site [46]. Therefore, the proximal poly(A) site is processed before the distal poly(A) site is transcribed, suggests a “first come, first served” mechanism. This resembles the extra domain I (EDI) alternative splicing mechanism described previously, whereby a slow RNAP II preferentially included the normally excluded alternative EDI exon because it allowed ample assembly time for the spliceosome machinery [36,47].

Eleven-nineteen lysine-rich leukemia gene (ELL) family proteins are essential components of the super elongation complex (SEC) and increase the catalytic rate of transcription elongation by RNA polymerase II [48–50]. ELL2 knockdown by siRNA affects the alternative pre-mRNA processing of the immunoglobulin heavy chain (IgH) gene, which is accompanied by reduced H3K4 and H3K79 methylation [50]. In addition to the ELLs, the SEC contains the MLL translocation partners AF4/FMR2 family member 1 (AFF1; also known as AF4), AFF4, eleven-nineteen leukemia (ENL) and ALL1-fused gene from chromosome 9 (AF9) [47]. In this complex, ENL is linked, not only with all members of the AF4 protein family that occur as MLL fusion partners, but also with pTEFb and DOT1L [51,52]. Notably, several frequent MLL fusion partners seem to coordinate DOT1L activity with a protein complex that stimulates the elongation phase of transcription by phosphorylating the carboxy-terminal repeat domain of RNA polymerase II [49,50].

H3K79me2 modification is tightly associated with active transcription [32,33]. Milcarek *et al.* speculated that conversion of monomethylated H3K79 into di- and trimethylated forms is correlated with the transition from low to high level gene transcription, due most likely to a decrease in the histone-DNA interaction [53]. Because H3K79 resides within the histone core, its methylation may facilitate DNA unwinding from the histone, allowing the downstream chromatin to open more readily and be transcribed more efficiently.

**Figure 6.**
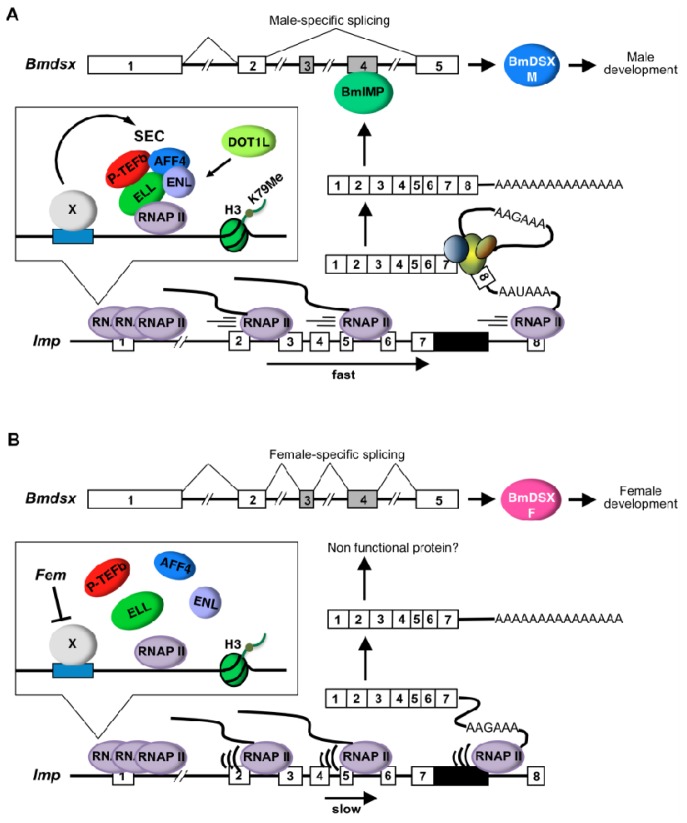
Model for the regulation of alternative splicing of *Imp* pre-mRNA by H3K79me2 and DOT1L. (**A**) In males, a transcription elongation factor (X) promotes the formation of SEC on *Imp*, causing H3K79 methylation by DOT1L. Higher RNAP II processivity due to H3K79me2 accumulation does not allow enough time to complete 3′-end processing at the proximal poly(A) site (AAGAAA), leading to exclusive use of the distal 3′ splice site of exon 8 and the distal poly(A) site (AAUAAA). While the distal poly(A) signal sequence is perfectly matched to the most canonical poly(A) signal hexamer, the proximal poly(A) signal sequence is consistent with a single-nucleotide variant of the canonical hexamer. Therefore, the polyadenylation machinery prefers the distal poly(A) site rather than the proximal poly(A) site when both poly(A) sites are present on the nascent transcript simultaneously. The protein product from the male-specific *Imp* transcript induces the male-specific splicing of *Bmdsx* pre-mRNA; (**B**) In females, the presence of a dominant feminizing factor, *Fem*, on the W chromosome directly or indirectly represses the expression of X, leading to failure of SEC formation. Decreased accumulation of H3K79me2 caused by the loss of SEC slows RNAP II processivity, providing sufficient time to recruit cleavage factors for 3′-end processing at the proximal poly(A) site. The absence of the male-specific Imp induces the female-specific splicing of *Bmdsx* pre-mRNA.

Based on these previous findings, we propose a possible model for the regulatory mechanism underlying sex-specific alternative splicing of *Imp* pre-mRNA (Figure 6). In male cells, exclusive expression of a transcription elongation factor (X), such as ELL family proteins, promotes the formation of SEC on *Imp*, causing H3K79 methylation by DOT1L. Higher RNAP II processivity due to H3K79me2 accumulation does not allow enough time to complete 3′-end processing at the proximal, non-sex-specific poly(A) site, leading to exclusive use of the 3′ splice site of exon 8. The male-specific Imp protein induces the male-specific splicing of *Bmdsx* pre-mRNA (Figure 6A). We have found recently that male-specific Imp bound immediately downstream of the proximal poly(A) site and promoted male-specific splicing of its pre-mRNA [28]. Therefore, after the male-specific Imp protein has been produced, the protein product may inhibit use of the proximal poly(A) site and promote the splicing of intron 7, leading to exclusive use of the 3′ splice site of exon 8. In female cells, *Fem* directly or indirectly represses the expression of X, leading to failure of SEC formation. Decreased accumulation of H3K79me2 caused by the loss of SEC slows RNAP II processivity, providing sufficient time to recruit cleavage factors such as CF I, CF II, and/or poly(A) polymerase (PAP) for 3′-end processing at the proximal poly(A) site. The absence of the male-specific Imp induces the female-specific splicing of *Bmdsx* pre-mRNA (Figure 6B).

In this model, both the proximal and distal poly(A) sites are exposed to the polyadenylation factors at the same time in male cells. How then is the distal poly(A) site exclusively selected? While the distal poly(A) signal sequence is perfectly matched to the most canonical poly(A) signal hexamer AAUAAA, the proximal poly(A) signal sequence is AAGAAA, which is consistent with a single-nucleotide variant of the canonical hexamer. The motifs that are functional in vertebrates are AAUAAA and its highly conserved variants (e.g., AUUAAA, UAUAAA, AGUAAA, AAGAAA) [54]. Among those hexamers, the canonical AAUAAA was reported to be present in 53% of the mRNAs; in contrast, a single-nucleotide variant AAGAAA was found in only 3% of the mRNA [55]. Therefore, one can reasonably suppose that the polyadenylation machinery prefers the distal poly(A) site rather than the proximal poly(A) site when both poly(A) sites are present on the nascent transcript simultaneously. This scenario is consistent with the kinetic coupling model for the regulation of alternative splicing by RNAP II elongation [56]. In this model, when a proximal suboptimal (weak) 3′ splice site and a downstream canonical (strong) 3′ splice site are presented simultaneously to the splicing machinery, the strong 3′ splice site could easily outcompete the weak site, resulting in alternative exon skipping. Our results indicate that both alternative polyadenylation and alternative splicing depend on RNAP II kinetics. In the present study, we were unable to assess precisely whether DOT1L plays a crucial role in regulating sex determination or sexual differentiation of *B. mori* because nearly all the embryos injected with *DOT1L* siRNA died before hatching. Consistent with our results, *Dot1L*-deficient embryos died between 9.5–10.5 days *post coitum* due to developmental abnormalities, including growth impairment, angiogenesis defects in the yolk sac, and cardiac dilation [57]. In *D. melanogaster*, *grappa* (*gpp*) is an ortholog of *Dot1L* [58]. *gpp* is an essential gene identified in a genetic screen for dominant suppressors of pairing-dependent silencing where a *Polycomb-group* (*Pc-G*)-mediated silencing mechanism necessary for the maintenance of parasegment identity during embryo development [59]. As is the case in *D. melanogaster*, *DOT1L* in *B. mori* may be required to maintain developmental gene expression through *Pc-G*-mediated mechanism.

Assuming that our model presented above is valid, an ELL family protein—such as ELL2—might be a key factor in facilitating male differentiation as a result of inducing the male-specific splicing of *Imp* pre-mRNA. Recently, we found that an *ELL2* homolog is expressed in embryos in early developmental stages. Ongoing investigations are aimed at determining whether the *ELL2* homolog is involved in regulating the sex-specific splicing of *Imp* pre-mRNAs.

## 3. Experimental Section

### 3.1. Silkworm Strains

The *Bombyx mori* non-diapausal and white egg strain (pnd-w1) was kindly provided by Kenichi Moto of RIKEN (Wako, Osaka, Japan). The S-2 strain, in which the females have the T (W; 2, 5) *p*^B^ + *^re^* (black egg, black larvae) genotype and the males have the + *^p^*^B^, *re* (red egg, white larvae) genotype, was established in our laboratory. The former strain was used primarily for RNAi experiments and the latter for ChIP-quantitative PCR (qPCR) and gene expression analysis. The developing eggs were enclosed in a plastic case and incubated at 25 ± 2 °C with sufficient humidity. Larvae were reared on an artificial diet (Nihon Nosan, Yokohama, Japan) at 25 ± 2 °C.

### 3.2. Preparation of siRNAs

**Figure 7.**
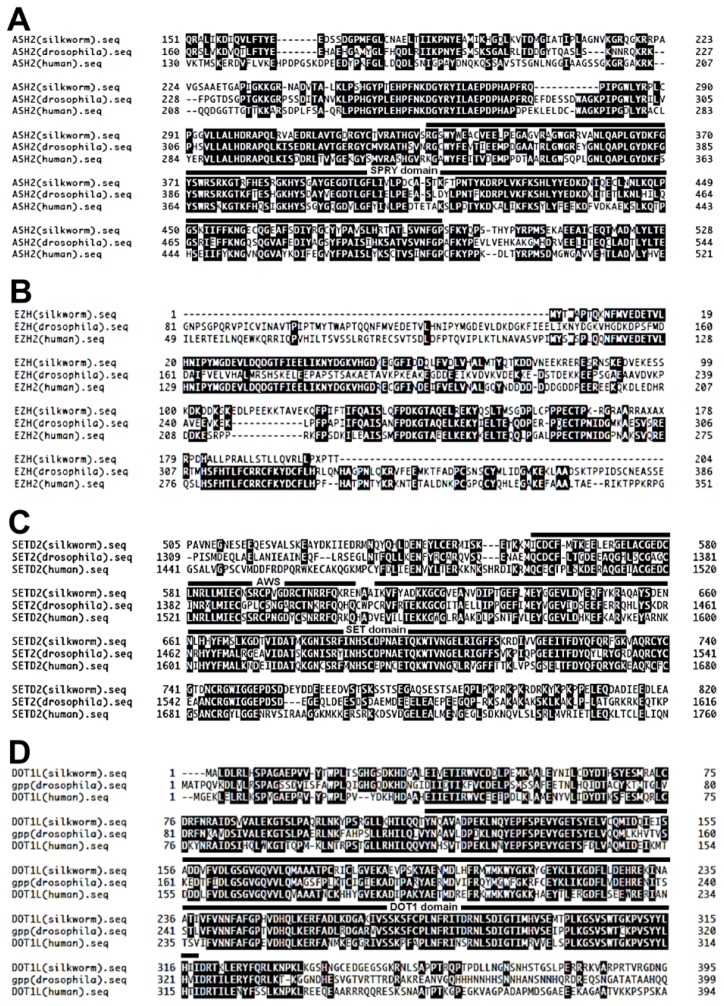
Predicted amino acid sequences of *Bombyx mori* ASH2, EZH2, SETD2, and DOT1L. (**A**) Alignment of *Bombyx mori* ASH2 (KAIKObase China Gene Model Gene No. BGIBMGA008025), *Drosophila melanogaster* ASH2, and human ASH2. The putative SPRY domain is denoted by horizontal line above the amino acid alignment; (**B**) Alignment of *Bombyx mori* EZH (KAIKObase EST clone ID: FS904534); (**C**) *Drosophila melanogaster* EZH, and human EZH; (**C**) Alignment of *Bombyx mori* SETD2 (KAIKObase China Gene Model Gene No. BGIBMGA003106), *Drosophila melanogaster* SETD2, and human SETD2. The putative AWS and SET domains are denoted by horizontal lines above the amino acid alignment; (**D**) Alignment of *Bombyx mori* DOT1L (KAIKObase FLcDNA clone name: ffbm34A09), *Drosophila melanogaster* Gpp (DOT1L ortholog), and human DOT1L. The putative DOT1 domain is denoted by horizontal line above the amino acid alignment. Amino acid identity is denoted by black boxes.

cDNA sequences predicted to encode ASH2, EZH2, SETD2, and DOT1L were retrieved by tBLASTn searches of the KAIKObase (http://sgp.dna.affrc.go.jp/KAIKObase/) using human ASH2, EZH2, SETD2, and DOT1L as query sequences (Figure 7). siRNA targeted to four HMTases (*ASH2*, *EZH2*, *SETD2*, *DOT1L*) were designed as described previously [60]. All sequences used in RNAi experiments are listed in Table 2. Each siRNA was synthesized using the custom select siRNA synthesis service provided by Ambion (Austin, TX, USA). Silencer negative control #1 siRNA (Ambion, Austin, TX, USA) was used as a negative control in the siRNA experiments.

### 3.3. Injection of siRNAs into Eggs

siRNAs were injected into eggs as described previously [60]. Negative control siRNA, *EZH2* siRNA, and *ASH2* siRNA were injected at 50 μM, and *SETD2* siRNA and *DOT1L* siRNA were injected at 50 or 100 μM.

### 3.4. Extraction of Total RNA and Genomic DNA

Total RNA was extracted from each egg and from NIAS-Bm-M1 cells using Isogen (Nippon Gene, Tokyo, Japan) according to the protocol described previously [60]. Genomic DNA was recovered by ethanol precipitation from the intermediate and organic phases obtained in the RNA extraction process. The precipitated DNA was purified using the SimplePrep^®^ reagent for DNA (Takara, Kyoto, Japan) according to the manufacturer’s instructions. To perform molecular sexing of each egg, PCR was performed using primers specific to the W chromosome genomic sequence according to the protocol described previously [60].

### 3.5. Reverse Transcription (RT)-PCR Analyses

RT-PCR reactions performed according to the protocol described previously [60]. The primer sequences and PCR conditions utilized in this study are indicated in Supplementary Table 3.

### 3.6. Quantitative Real-Time RT-PCR (qRT-PCR)

qRT-PCR assays were performed according to the protocol described previously [60]. All primer sequences used in this study are listed in Table 4. The BmEF-2F1 and BmEF-2R1 primers were used to amplify elongation factor-2 (*EF-2*) as an internal standard for quantification [61].

### 3.7. ChIP Experiments

The fifth instar day-3 larvae fat bodies were treated with 1× cold phosphate-buffered saline (PBS) containing 1% protease inhibitor cocktail (Roche, Basel, Switzerland). To cross-link samples, a formaldehyde solution (50 mM HEPES-KOH [pH 7.5], 100 mM NaCl, 1 mM EDTA [pH 8.0], 0.5 mM EGTA [pH 8.0], 11% formaldehyde) was added to a final concentration of 1% for 15 min at room temperature. Eggs were mashed with a pestle in the formaldehyde solution to prepare samples for ChIP experiments. The reaction was stopped by adding a glycine solution to a final concentration of 125 mM for 5 min at room temperature and washed twice with 1× cold PBS. The fixed fat bodies were disrupted with a Polytron (KINEMATICA, Lucern, Switzerland) (10,000 rpm, 20 s) and centrifuged at 2800 rpm for 5 min at 4 °C. The samples were resuspended in lysis buffer (10 mM Tris-HCl [pH 8.0], 100 mM NaCl, 1 mM EDTA [pH 8.0], 0.5 mM EGTA [pH 8.0], 0.1% Na-deoxycholate, 0.5% *N*-lauroylsarcosine, 1% protease inhibitor) and sonicated (pulsed 30 s, paused 1 min × 7 sets) on ice to avoid overheating. Triton-X was added to the resulting lysate at a final concentration of 1% and centrifuged at 15,000 rpm for 10 min at 4 °C. An aliquot (200 μL) of each sonicated sample served as an input DNA control. For ChIP, 300 μL of the supernatant was incubated with Dynabeads Protein G (Invitrogen, Carlsbad, CA, USA) with the appropriate antibody overnight at 4 °C. The beads were washed twice with 1× cold PBS. Next, 5 μg of antibodies were added in 300-μL blocking solution (0.5% bovine serum albumin [BSA]/PBS) and incubated overnight at 4 °C. The antibodies used in this study were anti-H3 antibody (ab1791, Abcam, Cambridge, UK), anti-H3K36me3 antibody (ab9050, Abcam, Cambridge, UK), anti-H3K79me2 antibody (ab3594, Abcam, Cambridge, UK), and anti-rabbit IgG antibody (12–370, Millipore, Billerica, MA, USA). The beads were washed once with low salt buffer (20 mM Tris-HCl [pH 8.0], 150 mM NaCl, 2 mM EDTA [pH 8.0], 0.1% SDS, 1% Triton X-100), twice with high salt buffer (20 mM Tris-HCl [pH 8.0], 400 mM NaCl, 2 mM EDTA [pH 8.0], 0.1% SDS, 1% Triton X-100), five times with RIPA buffer (50 mM HEPES-KOH [pH 7.5], 500 mM LiCl, 1 mM EDTA [pH 8.0], 1% NP-40, 0.7% Na-deoxycholate), and once with TE (Tris + EDTA) + 50 mM NaCl. To elute the histone-DNA complex, elution buffer (50 mM Tris-HCl [pH 8.0], 10 mM EDTA [pH 8.0], 1% SDS) was added to the beads and incubated at 65 °C for 15 min followed by centrifugation to obtain the supernatant. The supernatants were further incubated at 65 °C for 6 h, and then incubated with RNase A (Roche, Basel, Switzerland) at 37 °C for 2 h followed by incubation with proteinase K (20 mg/mL, Takara, Kyoto, Japan) at 55 °C for 2 h. Finally, the DNA was extracted with phenol chloroform and diluted in ddH_2_O. To calculate the amount of target sequence in the immunoprecipitated chromatin, we performed real-time qPCR as described above.

### 3.8. Mycophenolic Acid (MPA) and 6-Azauracil (6AU) Treatments

NIAS-Bm-M1 cells were maintained in IPL-41 (Invitrogen, Carlsbad, CA, USA) with 10% fetal bovine serum (FBS) (HyClone, Thermo Scientific, Waltham, MA, USA) under a humidifying atmosphere at 26 ± 1 °C and 2 mL of cultured cells were seeded onto a 35-mm dish. For MPA treatment, 2.5 μL of MPA (16 mg/mL in dimethyl sulfoxide (DMSO); Wako, Osaka, Japan) were added to each dish. For the 6AU treatment, 2.5 μL of 6AU (2 mg/mL in DMSO; Wako, Osaka, Japan) were added to each dish. The concentration of each inhibitor was determined as reported previously [43]. Total RNA was isolated at 1 or 3 days after treatment, respectively, according to the protocol described above.

## 4. Conclusions

Male-specific alternative splicing of *Imp* pre-mRNA was repressed by *DOT1L* depletion in male embryos. Consistent with this finding, higher distributions of H3K79me2 were observed in males than in females across *Imp*. Comparative analysis of RNAP II processivity indicated that RNAP II processivity was higher in males than in females at this locus. Inhibition experiments using inhibitors of RNAP II elongation suggested that the higher elongation rate was closely associated with male-specific splicing of *Imp* pre-mRNA. Taken together, our data suggest that greater accumulation of H3K79me2 along *Imp* in males causes increased RNAP II processivity, leading to male-specific alternative RNA processing in *Imp* mRNA production. Furthermore, knockdown of *DOT1L* caused embryonic lethality.

## Figures and Tables

**Table 1.** Effects of DOTIL siRNA injection on egg development.

siRNA	Injected eggs	Early or mid-stage embryonic lethal	Late-stage embryonic lethal	Viable
N. C. siRNA	115	58 (50.4%)	32 (27.8%)	25 (21.7%)
DOT1L siRNA	263	188 (71.5%)	74 (28.1%)	1 (0.4%) (male)

**Table 2.** siRNA sequences used in RNAi experiments.

Target gene	siRNA	Sense	Antisense
*SETD2*	393	UGCCAGCUCUGAGUCUGAUUCAAU	AUUGAAUCAGACUCAGAGCUGGCA
	490	CAGUGUAGCUCAAGAGAUATT	UAUCUCUUGAGCUACACUGTT
*DOT1L*	243	UUCCAAAGCAACUACAGAAUCGAUG	CAUCGAUUCUGUAGUUGCUUUGGAA
	353	AUUUACUCGCUUUACUUUGTT	CAAAGUAAAGCGAGUAAAUTT
*EZH2*	38	GACAACCCAACAGGUACCAAUAAGA	UCUUAUUGGUACCUGUUGGGUUGUC
	224	CGACGGGAAAGUGCAUGGUGAUAAA	UUUAUCACCAUGCACUUUCCCGUCG
*ASH2*	157	GACCGGCCUCUAGUCAAAUUCAAGA	UCUUGAAUUUGACUAGAGGCCGUC
	167	UAGUCAAAUUCAAGAGCCACCUGUA	UACAGGUGGCUCUUGAAUUUGACUA

**Table 3.** Primer sequences and PCR conditions utilized in this study.

Target gene	Primers	Sequence (5′ to 3′)	Denature	Annealing	Elongation	N° cycles
*Bmdsx*	FDSX-F2	CGCCTTACCGCAGACAGGCAG	98 °C	57 °C	57 °C	35
	FDSX-R4	GCGCAGTGTCGTCGCTACAAGG	10 s	30 s	60 s	
*Imp^M^*	BmIMPF1	ATGGACGGTGACATGTCTCAAG	98 °C	55 °C	57 °C	35
	BmIMPR1	TCATCCCGCCTCAGACGATTG	10 s	30 s	90 s	
*Imp^C^*	IMPE4F1	TCCCATAATAATCTCATTGGAC	98 °C	55 °C	57 °C	35
	IMPE7R1	AATGTGAACGGTGGTCTCGTG	10 s	30 s	90 s	
*Actin3*	BA3F1	AGATGACCCAGATCATGTTCG	98 °C	57 °C	57 °C	26
	BASR1	GAGATCCACATCTGTTGGAAG	10 s	30 s	30 s	
*m*	BmSxlF1	ATTAATCATCATAAAGCTACG	98 °C	57 °C	57 °C	35
	BmSxlR1	AATCCGTAACTGTAGCCAGTC	10 s	30 s	30 s	

**Table 4.** Sequences of primers used for qRT-PCR.

Target gene	Primers	Sequence (5′ to 3′)
*SETD2*	SETD2qPCRF1	CCTACAGGACATCTGGAGTTAC
	SETD2qPCRR1	GAATCAGTACCAGCATTTAGATG
*DOT1L*	dot1qPCRF1	AGAATCCGAACGACTCGACAG
	dot1qPCRR1	CTGTTCTTGGTCTTCGTTCAAC
*EZH2*	EZH2F1	GGTGTAGTGACAACCCAACAG
	EZH2R1	TCTTAACTCCTGAGCTGTTCC
*ASH2*	ASHF2	GGGGACCAGGTTCCACGAGTC
	ASHR2	TACAGGTGGCTCTTGAATTTG
*Bmdsx* promoter	BmdsxproF1	TGCATGTTTCTTATTAATCAGCTAG
	BmdsxproR1	GTAAATTTCGTAAAAGCTGACCAG
*Bmdsx* E1	ChIPEx1F	CCTGTACCACCAGTGGTGAAG
	ChIPEx1R	CTGACGGCGGTGGAGCGTATG
*Bmdsx* I2	ChIPI2F	CTACATGAACAGTACCAGTCAG
	ChIPI2R	GTAAGTACAAACTAAATAGCGTTC
*Bmdsx* E3	ChIPEx3F	GTCGACGAGTACGCGAGGAAG
	ChIPEx3R	TGTGATGCATGTATCTGTCGC
*Bmdsx* I3	ChIPI3F	GTAACTGACCTTCTTGCTAATC
	ChIPI3R	CTGTGCCATTTTATTAATATCGTC
*Bmdsx* E4	ChIPEx4F	ATATAAGTGGTGTACTGTCTTC
	ChIPEx4R	CCATAGATCCAATGTTACGAC
*Bmdsx* I4	ChIPI4F	GTTCAAACACATCGAAGCTAC
	ChIPI4R	GTCCGAGATAGACTGGCCTTG
*Bmdsx* E6	ChIPEx6F	GGCACAGCGCCGACAAGTAAG
	ChIPEx6R	ATTGTCTGTAGATATTCGTGATC
*BmIMP* promoter1S	IMPproF2	TTAAGCATTTAATTATAAGAAGATC
	IMPproR2	CTAGAATCTGCGATTACATAC
*BmIMP* E1S	IMPE1F1	TCCGTTCAGTACTCGCTATAC
	IMPE1R1	TCTTACCTATCGTCATAGATTC
*BmIMP* I1S	IMPI1F2	ATTTGGTAAAATAGTCTCGTATC
	IMPI1R2	ACCTTGTGATACGGGGTTAAC
*BmIMP* pro1L	IMPE1LproF1	GCTGCCCCACCCTTTAAACCG
	IMPE1LproR1	CTCGATCGTGCTGACTCTAGC
*BmIMP* E1L	IMPE1F1	TTTCAAGTATACTCCTTCTATAG
	IMPE1R1	TTCGCCATTTTGAGCAGATTG
*BmIMP* I1L	IMPI1F3	CAAATGGGCACATATTGTTGG
	IMPI1R3	GTTTAAGCGCTTTCGTGATGG
*BmIMP* E7	IMPE7F1	ATGCGGGAAGAAGGTTTTATG
	IMPE7R1	AATGTGAACGGTGGTCTCGTG
*BmIMP* I7	IMPI7F1	GTGCATAAATCCACAGAACAG
	IMPI7R1	TTACTCAGAAACTCAGAAGTAC
*BmIMP* E8	IMPE8F1	CGTCTGAGGCGGGATGAGAAC
	IMPE8R1	TAAATTCGCCGCAATCAGCAG
*EF1* promoter	EF-1proF1	TATATCAATTTTGGTGCAAGAATGG
	EF-1proR1	GTAATAATATTCTATTCTATCCACCG
*EF-1* E2	EF-1E2F1	TGGCGATGGAGGCGGAGAAG
	EF-1E2R1	CTCAACTTCCCAGCTGTCTGC
*EF-1* I3	EF-1I3F1	ACTTACTTATTTATGATCATGCGTC
	EF-1I3R1	GCTAACCACAATTATATTTGTGGAG
*EF-1* E6	EF-1E6F1	TACAGGTCATTTCTGCACGTAAG
	EF-1E6R1	TCATCCCAGTTAACTGTTGGATC
*EF-1* I11	EF-1I11F1	TTCATGGACTACATTTTACCTTGG
	EF-1I11R1	CTAAGCTCTTCTAAAAGAGATGAGC
*EF-1* I12	EF-1I12F1	GCATTAATATTAATTCCACCACAAG
	EF-1I12R1	CACACCTCACTGCTCTTCCGC
*Bm-Sxl* promoter	Sxli1F1	GGCTAAACTATCTTCAACAAG
	Sxli1R1	CGGTCACCGTTCTCGTGAAAG
*Bm-Sxl* E1	Sxle1F1	GCCAGTCCAAATGGACGAATC
	Sxle1R1	GTTCACTGACTTTCGAGTGAG
*Bm-Sxl* E2	Sxle2F1	GCCTACTCGAACAATAAAAAAG
	Sxle2R1	TTCCAAAGAATTGAAACTCCTG
*Bm-Sxl* I2	Sxli2F1	TGAATCAGAACATCTCATTTGG
	Sxli2R1	CCAAGCCGCTGCCTACCTAAC
*Bm-Sxl* E3	Sxle3F1	CGAGGCAGAGCGGGTTCGAAC
	Sxle3R1	CCTTCATCACTCGACAGCTCTC
*BmIMP* Proximal	IMPE2F1	ATCCTCAAAGGTACTCATCAG
	IMPE2I2R	GCATGCATCACTCAACAATAC
*BmIMP* Distal	IMPE7F2	GGATCATCGGCAAAGGCGGAC
	IMPI7R2	AGCACTTGGATCATTCATACC
*EF-1* Proximal	EF-1E2F1	TGGCGATGGAGGCGGAGAAGG
	EF-1E2I2R	GAAAAAGAAGAAAGCATTCATGC
*EF-1* Distal	EF-1I11E12F	ATGATACTGTATTAACTGCATTC
	EF-1E12R2	TTCAGGATTTTGAGACCCTGG
*BmIMP* E7-E8	IMPE7F1	ATGCGGGAAGAAGGTTTTATG
	BmIMPR1	TCATCCCGCCTCAGACGATTG
*BmIMP* E1-E3	BmIMPF1	ATGGACGGTGACATGTCTCAAG
	IMPE3R1	CATCCATTCAACCCGTTTATG
*BmIMP* E1S	BmIMPF1	ATGGACGGTGACATGTCTCAAG
	IMPE2R2	GCCTGCTCTGGACTCTCGAAG
*BmIMP* E1L	IMPE1LF1	AATCTGCTCAAAATGGCGAAG
	IMPE2R2	GCCTGCTCTGGACTCTCGAAG
*Bm-Sxl* S	BmSxlF2	ACTCGCGTTACCTATTTAAC
	BmSxlSR1	GTACTGCTGTTGGATTTGGTC
*Bm-Sxl* L	BmSxlF2	ACTCGCGTTACCTATTTAAC
	BmSxlLR1	CTGCTGTTGGATTTGATTTTC

## References

[1] Bandziulis R.J., Swanson M.S., Dreyfuss G. (1989). RNA-binding proteins as developmental regulators. Genes Dev.

[2] Green M.R. (1991). Biochemical mechanisms of constitutive and regulated pre-mRNA splicing. Annu. Rev. Cell Biol.

[3] Wang E.T., Sandberg R., Luo S., Khrebtukova I., Zhang L., Mayr C., Kingsmore S.F., Schroth G.P., Burge C.B. (2008). Alternative isoform regulation in human tissue transcriptomes. Nature.

[4] Black D.L. (1995). Finding splice sites within a wilderness of RNA. RNA.

[5] Chou M.Y., Rooke N., Turck C.W., Black D.L. (1999). hnRNP H is a component of a splicing enhancer complex that activates a c-src alternative exon in neuronal cells. Mol. Cell. Biol.

[6] Expert-Bezancon A., Le Caer J.P., Marie J. (2002). Heterogeneous nuclear ribonucleoprotein (hnRNP) K is a component of an intronic splicing enhancer complex that activates the splicing of the alternative exon 6A from chicken beta-tropomyosin pre-mRNA. J. Biol. Chem.

[7] Li X., Shambaugh M.E., Rottman F.M., Bokar J.A. (2000). SR proteins Asf/SF2 and 9G8 interact to activate enhancer-dependent intron D splicing of bovine growth hormone pre-mRNA*in vitro*. RNA.

[8] Modafferi E.F., Black D.L. (1999). Combinatorial control of a neuron-specific exon. RNA.

[9] Alló M., Schor I.E., Muñoz M.J., de la Mata M., Agirre E., Valcárcel J., Eyras E., Kornblihtt A.R. (2010). Chromatin and alternative splicing. Cold Spring Harb. Symp. Quant. Biol.

[10] Luco R.F., Allo M., Schor I.E., Kornblihtt A.R., Misteli T. (2011). Epigenetics in alternative pre-mRNA splicing. Cell.

[11] Luco R.F., Misteli T. (2011). More than a splicing code: Integrating the role of RNA, chromatin and non-coding RNA in alternative splicing regulation. Curr. Opin. Genet. Dev.

[12] Spies N., Nielsen C.B., Padgett R.A., Burge C.B. (2009). Biased chromatin signatures around polyadenylation sites and exons. Mol. Cell.

[13] Schwartz S., Meshorer E., Ast G. (2009). Chromatin organization marks exon-intron structure. Nat. Struct. Mol. Biol.

[14] Tilgner H., Nikolaou C., Althammer S., Sammeth M., Beato M., Valcárcel J., Guigó R. (2009). Nucleosome positioning as a determinant of exon recognition. Nat. Struct. Mol. Biol.

[15] Schor I.E., Rascovan N., Pelisch F., Alló M., Kornblihtt A.R. (2009). Neuronal cell depolarization induces intragenic chromatin modifications affecting *NCAM* alternative splicing. Proc. Natl. Acad. Sci. USA.

[16] Kolasinska-Zwierz P., Down T., Latorre I., Liu T., Liu X.S., Ahringer J. (2009). Differential chromatin marking of introns and expressed exons by H3K36me3. Nat. Genet.

[17] Luco R.F., Pan Q., Tominaga K., Blencowe B.J., Pereira-Smith O.M., Misteli T. (2010). Regulation of alternative splicing by histone modifications. Science.

[18] Sims R.J., Millhouse S., Chen C.F., Lewis B.A., Erdjument-Bromage H., Tempst P., Manley J.L., Reinberg D. (2007). Recognition of trimethylated histone H3 lysine 4 facilitates the recruitment of transcription postinitiation factors and pre-mRNA splicing. Mol. Cell.

[19] Saint-André V., Batsché E., Rachez C., Muchardt C. (2011). Histone H3 lysine 9 trimethylation and HP1γ favor inclusion of alternative exons. Nat. Struct. Mol. Biol.

[20] Zhou Y., Lu Y., Tian W. (2012). Epigenetic features are significantly associated with alternative splicing. BMC Genomics.

[21] Hashimoto H. (1933). The role of the W chromosome for sex determination in the silkworm*Bombyx mori*. Jpn. J. Genet.

[22] Niimi T., Sahara K., Oshima H., Yasukouchi Y., Ikeo K., Traut W. (2006). Molecular cloning and chromosomal localization of the *Bombyx Sex-lethal* gene. Genome.

[23] Mita K., Kasahara M., Sasaki S., Nagayasu Y., Yamada T., Kanamori H., Namiki N., Kitagawa M., Yamashita H., Yasukochi Y. (2009). The genome sequence of silkworm*Bombyx mori*. DNA Res.

[24] Suzuki M.G., Funagume S., Kanda T., Tamura T., Shimada T. (2005). Role of the male BmDSX protein in the sexual differentiation of*Bombyx mori*. Evol. Dev.

[25] Suzuki M.G., Ohbayashi F., Mita K., Shimada T. (2001). The mechanism of sex-specific splicing at the *doublesex* gene is different between *Drosophila melanogaster* and*Bombyx mori*. Insect Biochem. Mol. Biol.

[26] Suzuki M.G., Imanishi S., Dohmae N., Nishimura T., Shimada T., Matsumoto S. (2008). Establishment of a novel *in vivo* sex-specific splicing assay system to identify a trans-acting factor that negatively regulates splicing of *Bombyx mori dsx* female exons. Mol. Cell. Biol.

[27] Suzuki M.G., Imanishi S., Dohmae N., Asanuma M., Matsumoto S. (2010). Identification of a male-specific RNA binding protein that regulates sex-specific splicing of *Bmdsx* by increasing RNA binding activity of BmPSI. Mol. Cell. Biol.

[28] Suzuki M.G., Kobayashi S., Aoki F. (2014). Male-specific splicing of the silkworm *Imp* gene is maintained by an autoregulatory mechanism. Mech. Dev.

[29] Terenius O., Papanicolaou A., Garbutt J.S., Eleftherianos I., Huvenne H., Kanginakudru S., Albrechtsen M., An C., Aymeric J.L., Barthel A. (2011). RNA interference in Lepidoptera: An overview of successful and unsuccessful studies and implications for experimental design. J. Insect Physiol.

[30] Yamaguchi J., Mizoguchi T., Fujiwara H. (2011). siRNAs induce efficient RNAi response in *Bombyx mori*. PLoS One.

[31] Nguyen A.T., Zhang Y. (2011). The diverse functions of Dot1 and H3K79 methylation. Genes Dev.

[32] Schübeler D., MacAlpine D.M., Scalzo D., Wirbelauer C., Kooperberg C., van Leeuwen F., Gottschling D.E., O’Neill L.P., Turner B.M., Delrow J. (2004). The histone modification pattern of active genes revealed through genome-wide chromatin analysis of a higher eukaryote. Genes Dev.

[33] Morillon A., Karabetsou N., Nair A., Mellor J. (2005). Dynamic lysine methylation on histone H3 defines the regulatory phase of gene transcription. Mol. Cell.

[34] Pokholok D.K., Harbison C.T., Levine S., Cole M., Hannett N.M., Lee T.I., Bell G.W., Walker K., Rolfe P.A., Herbolsheimer E. (2005). Genome-wide map of nucleosome acetylation and methylation in yeast. Cell.

[35] Kadener S., Fededa J.P., Rosbash M., Kornblihtt A.R. (2002). Regulation of alternative splicing by a transcriptional enhancer through RNA pol II elongation. Proc. Natl. Acad. Sci. USA.

[36] De la Mata M., Alonso C.R., Kadener S., Fededa J.P., Blaustein M., Pelisch F., Cramer P., Bentley D., Kornblihtt A.R. (2003). A slow RNA polymerase II affects alternative splicing*in vivo*. Mol. Cell.

[37] Nakanishi T., Nakano A., Nomura K., Sekimizu K., Natori S. (1992). Purification, gene cloning, and gene disruption of the transcription elongation factor S-II in*Saccharomyces cerevisiae*. J. Biol. Chem.

[38] Nakanishi T., Shimoaraiso M., Kubo T., Natori S. (1995). Structure-function relationship of yeast S-II in terms of stimulation of RNA polymerase II, arrest relief, and suppression of 6-azauracil sensitivity. J. Biol. Chem.

[39] Powell W., Reines D. (1996). Mutations in the second largest subunit of RNA polymerase II cause 6-azauracil sensitivity in yeast and increased transcriptional arrest *in vitro*. J. Biol. Chem..

[40] Franklin T.J., Cook J.M. (1969). The inhibition of nucleic acid synthesis by mycophenolic acid. Biochem. J.

[41] Exinger F., Lacroute F. (1992). 6-Azauracil inhibition of GTP biosynthesis in*Saccharomyces cerevisiae*. Curr. Genet.

[42] Uptain S.M., Kane C.M., Chamberlin M.J. (1997). Basic mechanisms of transcript elongation and its regulation. Annu. Rev. Biochem.

[43] Howe K.J., Kane C.M., Ares M. (2003). Perturbation of transcription elongation influences the fidelity of internal exon inclusion in*Saccharomyces cerevisiae*. RNA.

[44] Masumoto M., Yaginuma T., Niimi T. (2009). Functional analysis of *Ultrabithorax* in the silkworm, *Bombyx mori*, using RNAi. Dev. Genes Evol.

[45] Llamazares S., Moreira A., Tavares A., Girdham C., Spruce B.A., Gonzalez C., Karess R.E., Glover D.M., Sunkel C.E. (1991). *polo* encodes a protein kinase homolog required for mitosis in*Drosophila*. Genes Dev.

[46] Pinto P.A., Henriques T., Freitas M.O., Martins T., Domingues R.G., Wyrzykowska P.S., Coelho P.A., Carmo A.M., Sunkel C.E., Proudfoot N.J. (2011). RNA polymerase II kinetics in *poilo* polyadenylation signal selection. EMBO J.

[47] De la Mata M., Lafaille C., Kornblihtt A.R. (2010). First come, first served revisited: Factors affecting the same alternative splicing event have different effects on the relative rates of intron removal. RNA.

[48] Luo Z., Lin C., Shilatifard A. (2012). The super elongation complex (SEC) family in transcriptional control. Nat. Rev. Mol. Cell Biol.

[49] Shilatifard A., Lane W.S., Jackson K.W., Conaway R.C., Conaway J.W. (1996). An RNA polymerase II elongation factor encoded by the human ELL gene. Science.

[50] Shilatifard A., Duan D.R., Haque D., Florence C., Schubach W.H., Conaway J.W., Conaway R.C. (1997). ELL2, a new member of an ELL family of RNA polymerase II elongation factors. Proc. Natl. Acad. Sci. USA.

[51] Bitoun E., Oliver P.L., Davies K.E. (2007). The mixed-lineage leukemia fusion partner AF4 stimulates RNA polymerase II transcriptional elongation and mediates coordinated chromatin remodeling. Hum. Mol. Gen.

[52] Mueller D., Bach C., Zeisig D., Garcia-Cuellar M.P., Monroe S., Sreekumar A., Zhou R., Nesvizhskii A., Chinnaiyan A., Hess J.L. (2007). A role for the MLL fusion partner ENL in transcriptional elongation and chromatin modification. Blood.

[53] Milcarek C., Albring M., Langer C., Park K.S. (2011). The eleven-nineteen lysine-rich leukemia gene (ELL2) influences the histone H3 protein modifications accompanying the shift to secretory immunoglobulin heavy chain mRNA production. J. Biol. Chem.

[54] Colgan D.F., Manley J.L. (1997). Mechanism and regulation of mRNA polyadenylation. Genes Dev.

[55] Tian B., Hu J., Zhang H., Lutz C.S. (2005). A large-scale analysis of mRNA polyadenylation of human and mouse genes. Nucleic Acids Res.

[56] Kornblihtt A.R. (2006). Chromatin, transcript elongation and alternative splicing. Nat. Struct. Mol. Biol.

[57] Jones B., Su H., Bhat A., Lei H., Bajko J., Hevi S., Baltus G.A., Kadam S., Zhai H., Valdez R. (2008). The histone H3K79 methyltransferase Dot1L is essential for mammalian development and heterochromatin structure. PLoS Genet.

[58] Shanower G.A., Muller M., Blanton J.L., Honti V., Gyurkovics H., Schedl P. (2005). Characterization of the *grappa* gene, the *Drosophila* histone H3 lysine 79 methyltransferase. Genetics.

[59] Brock H.W., van Lohuizen M. (2001). The *Polycomb* group—No longer an exclusive club. Curr. Opin. Genet. Dev.

[60] Suzuki M.G., Suzuki K., Aoki F., Ajimura M. (2012). Effect of RNAi-mediated knockdown of the *Bombyx mori transformer-2* gene on the sex-specific splicing of *Bmdsx* pre-mRNA. Int. J. Dev. Biol.

[61] Koike Y., Mita K., Suzuki M.G., Maeda S., Abe H., Osoegawa K., deJong P.J., Shimada T. (2003). Genomic sequence of a 320-kb segment of the Z chromosome of *Bombyx mori* containing a *kettin* ortholog. Mol. Genet. Genomics.

